# Health-Promoting Properties and the Use of Fruit Pomace in the Food Industry—A Review

**DOI:** 10.3390/nu16162757

**Published:** 2024-08-18

**Authors:** Ewa Raczkowska, Paweł Serek

**Affiliations:** Department of Human Nutrition, Faculty of Biotechnology and Food Science, Wroclaw University of Environmental and Life Sciences, 37 Chelmonskiego Street, 51-630 Wroclaw, Poland; pawel.serek@upwr.edu.pl

**Keywords:** fruit pomace, antioxidant, anti-diabetic, anti-inflammatory, antibacterial, food industry, bread, sweet snack products, extruded snacks

## Abstract

Fruit pomace, a by-product of the fruit industry, includes the skins, seeds, and pulp most commonly left behind after juice extraction. It is produced in large quantities: apple residues alone generate approximately 4 million tons of waste annually, which is a serious problem for the processing industry but also creates opportunities for various applications. Due to, among other properties, their high content of dietary fiber and polyphenolic compounds, fruit residues are used to design food with functional features, improving the nutritional value and health-promoting, technological, and sensory properties of food products. This article presents the health-promoting (antioxidant, antidiabetic, anti-inflammatory, and antibacterial) properties of fruit pomace. Moreover, the possibilities of their use in the food industry are characterized, with particular emphasis on bread, sweet snack products, and extruded snacks. Attention is paid to the impact of waste products from the fruit industry on the nutritional value and technological and sensory characteristics of these products. Fruit pomace is a valuable by-product whose use in the food industry can provide a sustainable solution for waste management and contribute to the development of functional food products with targeted health-promoting properties.

## 1. Introduction

The fruit industry generates approximately 50% of its by-products in the form of peels, seeds, and pomace after pressing juices and damaged and immature raw materials [1,2]. The production volume of fruit pomace varies depending on the specific fruit. The most frequently mentioned volume in the literature is the production of apple pomace, which is approximately 4 million tons per year and is produced as a result of the production of apple juice [3]. In the case of apple pomace, its production in the years 1994–2021 increased from 46 to 86 million tons, with global production of apple juice at the level of over 4.5 million tons per year [1]. These figures highlight the significant volume of fruit pomace produced by the fruit-processing industry and the potential to use this by-product for a variety of applications. Waste products from the fruit industry can pose a risk if not managed properly. The improper management of fruit pomace can lead to environmental pollution and public health risks due to the risk of microbial contamination [4]. However, research results prove that fruit pomace has many potential applications. They can be used, among other things, as a source of organic matter for the soil, improving its properties and increasing crop yields [5]. Fruit pomace such as apple, grape, blackcurrant, and orange pomace can also be used as a biosorbent to remove heavy metal ions from aqueous solutions [6]. Additionally, fruit pomace contains biologically active compounds that have, among other things, antibacterial properties, making it suitable for use in food and packaging production [7]. Moreover, fruit pomace, due to its high content of dietary fiber and polyphenol compounds, is used as a functional food ingredient and to formulate recipes for food products with targeted health-promoting, technological, and sensory properties [8,9,10]. Therefore, when properly managed and used, fruit pomace can be a valuable resource for various industries, contributing to sustainable development and having a positive economic impact in the future.

Therefore, this literature review aimed to present the antioxidant, antidiabetic, anti-inflammatory, and antibacterial properties of pomace from various fruits. Moreover, the possibility of using fruit pomace to produce bread, sweet snacks, and extruded products was presented, paying attention to their impact on the nutritional value, technological features, and sensory values of the final products.

## 2. Article Search Methodology

The article uses scientific literature published in English. The following databases were used to search for articles: Web of Science, Scopus, and Google Scholar. Keywords used in the search were: fruit pomace AND bread OR sweet snack products OR extruded snacks. The manual selection of literature entries was sufficient to obtain an adequate amount of data. Articles used in this review were published between January 2004 and June 2024. To prepare a table presenting the description of the use of fruit pomace in bread production, 21 articles were used; sweet snack products—29; and extruded products—17.

## 3. Health-Promoting Properties of Fruit Pomace

### 3.1. Antioxidant Properties

Researchers are conducting numerous studies on the possibilities of using waste raw materials from the fruit and vegetable industry, focusing, among other things, on designing recipes for food products with functional characteristics that benefit human health [11,12,13,14]. Fruit pomace has antioxidant properties due to the significant content of polyphenol compounds. The appropriate concentration and location of reactive oxygen species in cells are crucial for preventing oxidative damage to nucleic acids, proteins, and fats [15]. Additionally, they can act as reactive oxygen scavengers and free-radical inhibitors [16]. Studies involving animals show that adding fruit pomace to feed affects the antioxidant activity of their blood plasma. Rodríguez-Muela et al. (2015) showed that the blood plasma of lambs supplemented with fermented apple pomace in feed at the level of 10.91% was characterized by a significantly higher antioxidant activity (24.34 vs. 21.79 mM Fe2, *p* < 0.06) and an increased number of leukocytes compared to animals from the control group (from 7.52 × 10^3^ ± 1.29 × 10^3^ μL to 9.14 × 10^3^ ± 1.24 × 10^3^ μL, *p* < 0.05) [17]. Similar conclusions were reached in a study by Juśkiewicz et al. (2012) conducted on Wistar rats. The animals were fed apple pomace, and the activity of superoxide dismutase in their erythrocytes was increased (from 235 ± 7 to 281 ± 14 mmol/min per L) [18]. Human studies on the antioxidant properties of fruit waste also show promising results. Radić et al. (2020) showed that olive pomace extract had a strong antioxidant effect on human HepG2 cells. The protective effect against oxidative stress consisted of increasing the intracellular glutathione concentration and reducing the accumulation of reactive oxygen species [19]. In turn, in a study by Choleva et al. (2022), grape pomace extract showed an antioxidant effect in healthy women after a high-fat meal, reducing markers of oxidative stress, such as uric acid (from 4.1 ± 0.8 to 3.6 ± 1.0 mg/dL) and thiobarbituric acid (from 2.8 ± 2.0 to 2.2 ± 0.8 μM) reactive substances, and increasing the activity of superoxide dismutase (from 10.9 ± 4.1 to 11.1 ± 4.5 U/mg pr) [20]. Pili fruit pomace, which was served to healthy people as an addition to the drink, also had health-promoting properties. Significant changes were observed in the total antioxidant capacity (determined using the ferric-ion-reducing antioxidant parameter—FRAP) and the total polyphenol content (determined using the Folin–Ciocalteu method) in the subjects’ blood plasma. Maximum levels were reached at 120 and between 30 and 60 min of the test, respectively (*p* < 0.05). Additionally, both the concentration of polyphenolic compounds in plasma and the total antioxidant capacity remained significantly above baseline values throughout the study period (*p* < 0.05) [21].

In turn, in one of the in vitro studies, various tests were carried out to assess the antioxidant capacity of phenolic compounds found in pomegranate peel. The IC50 values for DPPH free-radical scavenging activity, the ABTS assay, and lipid peroxidation inhibitory activity were only 0.1 µg/mL. The same for hydroxyl free-radical scavenging activity was 0.6 µg/mL. The nitric oxide scavenging activity had the lowest IC50 value (0.02 µg/mL). At a concentration of 0.21 µg/mL, phenols scavenged approximately 60% of DPPH radicals. In turn, with a phenol content of 98.9 µg/mL, the extract chelated 60% of metallic iron. Based on various tests, it has been shown that pomegranate peel extract has significant antioxidant activity and can be used as a source of natural antioxidants in the design of functional foods and nutraceuticals [22]. The available literature relatively extensively discusses the antioxidant properties of apple pomace, which largely depend on the fruit variety. One study showed that Gala apple pomace has an approximately 3-fold greater ability to capture 1-diphenyl-2-picrylhydrazyl (DPPH) radical scavenging and even a nearly 30-fold greater ability to capture superoxide scavenging compared to vitamins C and E [23]. Another study compared the properties of pomace from six Spanish varieties. It was shown that all varieties were a source of polyphenolic compounds, but their antioxidant properties varied. Pomace from the Carrio variety was characterized by the highest DPPH (15.9 vs. 11.1–13.5 g AA/kg) and FRAP (13.8 vs. 9.5–18.8 g AA/kg) values (*p* < 0.05), while the de la Riega, Limon Montes, and Meana varieties were characterized by higher DPPH (13.5, 11.1 and 12.4 g AA/kg, respectively) values compared to the other varieties. Additionally, the authors pointed out that a higher antioxidant activity is associated with a higher content of phloridzins, procyanidin B2, rutin + isoquercitrin, protocatechuic acid, and hyperin [24].

The above research suggests that including fruit pomace in the diet may positively impact metabolic responses and overall health, making them valuable sources of natural antioxidants for potential use in functional foods or nutraceutical products.

### 3.2. Anti-Diabetic Properties

Due to the high content of bioactive compounds, fruit pomace is often tested for its potential antidiabetic effects. Moreover, it is emphasized that using fruit pomace to produce natural antioxidant and antidiabetic extracts rich in polyphenols and flavonoids is a sustainable alternative in the treatment of diabetes and related metabolic disorders. Their hypoglycemic effect was tested during intervention studies, which differed in the methodology, size of the study group, duration of the study source of the obtained pomace, and method of its administration and inclusion in the diet.

A significant amount of research has been conducted on orange pomace. Guzman et al. (2021) assessed the effect of orange pomace on the postprandial glycemic response in healthy people. The study aimed to determine whether adding fruit pomace to orange juice could reduce the glycemic response and whether the results differed from the effect of consuming the whole fruit. Two studies assessed the 2-h glucose level curve (AUC) and maximum glucose concentration (Cmax). The results showed that juice with added pomace did not significantly reduce the AUC compared to juice without added pomace or whole fruit (825 ± 132 compared with 920 ± 132 and 760 ± 132 mg · min · dL^−1^, respectively, *p* = 0.57 for both), but significantly lowered the glucose Cmax in both studies (study 1:5 g fiber from orange pomace, 115 ± 4.06 compared with orange juice, 124 ± 4.06 and whole orange fruit, 114 ± 4.06 mg · dL^−1^, *p* = 0.002 and 0.75, respectively; study 2:5 g fiber from orange pomace, 128 ± 1.92 compared with orange juice, 136 ± 1.92 and whole orange fruit, 125 ± 1.92 mg · dL^−1^, *p* = 0.001 and 0.28, respectively). The study also showed that the Cmax of insulin tended to be lower after whole fruit consumption compared to that of pomace-containing juice [25]. Another study examined the effects of adding enzyme-treated orange pomace to orange juice (100% orange juice or 100% orange juice with 5 g of enzyme-treated orange pomace fiber). The pomace fiber reduced the glucose availability by delaying gastric emptying, binding glucose, and increasing food transit. The AUC and Cmax of glucose were significantly reduced in the study group (48.5 ± 15.3 vs. 13.5 ± 12.7 mmol min L^−1^, *p* = 0.02 and 7.2 ± 0.9 vs. 6.5 ± 0.8 mmol L^−1^, *p* < 0.001, respectively); however, the AUC and Cmax of insulin showed no significant differences. The study also showed that pomace fiber subjected to enzymatic treatment to reduce the viscosity of the raw material retained its glucose-lowering effect [26]. The effect of high-fiber orange pomace on the postprandial glycemic response in overweight men was also investigated. Participants consumed a placebo, a small dose of a pomace drink (35%), or a large dose of a pomace drink (77%) with breakfast, and their blood glucose and insulin levels were measured over a 2 h period. The results showed that drink consumption delayed the time to peak serum glucose levels (from 33 (placebo) to 45 (large dose of a pomace drink) and 47 min (small dose of a pomace drink) (*p* = 0.055 and 0.013, respectively)) and reduced post-breakfast insulin levels. A high dose of pomace reduced the area under the insulin concentration curve (AUC) in the first 2 h (by 23% compared to placebo) and attenuated the glucose and insulin concentrations in the next meal [27]. Dong et al. (2016) studied men with an increased cardiometabolic risk. Participants were randomly assigned to one of four groups at each intervention: a group drinking pure orange juice without the pulp, a juice with added orange fiber, a juice made from gently blended whole oranges, and a control group. The results showed that the addition of orange fibers significantly (*p* < 0.05) reduced the maximal change in glucose concentrations (1.9 ± 0.21 mmol/L) reached after breakfast compared with other treatments (2.3–2.4 mmol/L) and after lunch (3.0 ± 0.05 mmol/L) compared with orange juice (3.6 ± 0.05 mmol/L). The maximal change in insulin concentration (313 ± 25 pmol/L) was also lower compared with control (387 ± 30 pmol/L) and orange juice (418 ± 39 pmol/L) after breakfast. It also delayed the time to reach the maximum glucose and insulin concentrations. Other studies by Dong et al. (2016) showed that adding fiber to orange juice increased short-term satiety, which may be a natural and effective way to control appetite and reduce food intake [28,29].

Red grape pomace is another fruit often evaluated for its antidiabetic potential. Campos et al. (2021) examined polyphenols and polysaccharide–polyphenol complexes extracted from such pomace. The study assessed the antidiabetic effects of these substances, including the inhibition of carbohydrate metabolizing enzymes, antiglycation effects, and glucose uptake by Caco-2 cell monolayers. A dose-dependent inhibitory effect on α-amylase and α-glucosidase was found, and the effect varied depending on the fraction from which the extract was obtained. Free polyphenols with hydroxyl groups can interfere with substrate–enzyme binding through various interactions, leading to enzyme inhibition, and the extracts showed a more pronounced inhibitory effect on α-glucosidase than α-amylase. Polyphenols such as epicatechin, cyanidin, ferulic acid, caffeic acid, quercetin, and syringic acid showed a high affinity for the active sites of α-glucosidase. Polysaccharide–polyphenol conjugates showed antidiabetic potential, although lower than that of free polyphenols. Polyphenolic compounds associated with branched-chain polysaccharides were able to weaken glucose transport through monolayers of human Caco-2 cells and showed a greater ability to reduce α-amylase activity; at the same time, oxidized polyphenol structures had a greater impact on α-glucosidase activity [30].

In a pilot study, Costabile et al. (2019) examined the acute effect of consuming a drink rich in red grape pomace polyphenols on the blood concentration of glucose, insulin, and triglycerides after healthy people consumed a standard meal. The study found that drink consumption lowered postprandial insulin levels and improved insulin sensitivity. There was no effect on glucose and triglyceride levels. The area under the insulin secretion curve was 31% lower (*p* < 0.05), insulin secretion was 18% lower (*p* < 0.016), and the insulin sensitivity index was 36% higher (*p* = 0.037) after subjects consumed the test drink versus the control drink. The plasma concentration of gallic acid, a phenolic metabolite, was inversely correlated with the postprandial insulin response (r = −0.604; *p* = 0.032) and positively correlated with the insulin sensitivity index (r = 0.588, *p* = 0.037). The experiment also examined the relationship of polyphenol intake to metabolic reactions and the pharmacokinetic parameters of phenolic metabolites detected in plasma [31]. The results provide information needed to understand the acute effects of grape polyphenols on insulin sensitivity and glucose metabolism in healthy individuals. A clinical study was also conducted to determine the effect of grape pomace supplementation in people at risk of cardiovascular diseases on glucose homeostasis, cardiometabolic risk factors, biochemical parameters of iron metabolism, anthropometric measurements, and intestinal transit. The study found that grape pomace supplementation significantly improved fasting insulinemia and insulin sensitivity (*p* < 0.01). However, there was no significant effect on other parameters such as lipid profiles, blood pressure, iron levels, and anthropometric measurements. Additionally, the subjective experience indicated improved intestinal transit after supplementation with grape pomace [32]. The study suggests that grape pomace intake may improve insulin sensitivity in people with a high cardiometabolic risk.

Perez-Ramirez et al. (2020) assessed the effects of a grape pomace and pomegranate dietary supplement on glucose metabolism and oxidative stress in adults with abdominal obesity. The study design included the administration of a standard 75 g oral glucose tolerance test (OGTT) with or 10 h after dietary supplement intake. The dietary supplement used contained both extracted (0.4 g) and unextracted (1.4 g) polyphenols. The area under the curve (AUC) for glucose was examined 0–120 min after OGTT. The concentration of insulin and uric acid as well as the oxidative state of the body were also measured. The study found that the dietary supplement did not significantly improve glucose or insulin levels at any sampling point. It also showed no improvement in antioxidant capacity in plasma or urine, and no significant increase in urinary polyphenol excretion was observed. However, a trend towards improved insulin sensitivity was observed in the group in which the dietary supplement was consumed 10 h before the glucose solution, suggesting a potential effect on insulin sensitivity with long-term intake [33].

The possible antihyperglycemic effects of a preparation (25 g) containing phlorizin obtained from unripe apple pomace were also investigated in healthy volunteers. The apple preparation was found to reduce postprandial glycemia and increase urinary glucose excretion (during the 0 to 2 h and the 2 to 4 h interval of the OGTT by approximately 20-fold and 5-fold, respectively) during oral glucose tolerance tests in six healthy volunteers. The study identified and quantified phlorizin metabolites in the blood and urine of people after they consumed the apple preparation. The results indicated a relationship between the concentration of phlorizin metabolites in urine and the effect of glycosuria after the participants consumed the apple preparation [34]. However, studies indicate that phlorizin is rapidly metabolized, which may limit its bioavailability and therapeutic efficacy. Ohta et al. (2012) emphasize that phlorizin undergoes significant metabolic transformation in the liver, which leads to reduced concentration in the systemic circulation [35]. Other authors point to the role of specific enzymes in the metabolism of phlorizin, suggesting that the genetic variability of these enzymes may influence individual responses to the compound [36]. It is also emphasized that phlorizin should not be used in humans due to its toxicity and low bioavailability [37,38].

Fruit pomace is being investigated as a possible raw material for confectionery, as an ingredient that reduces the postprandial increase in blood glucose. Consuming muffins enriched with sour cherry pomace (up to 30%) reduced the postprandial glycemic response compared to consuming a muffin without pomace (with cherry-pomace-treated muffins, glucose responses were significantly lower at 30, 45, and 60 min intervals, and the incremental peak glucose was 0.40 and 0.60 mmol L^−1^ lower than for control muffins). The addition also increased the satiating effect of the muffins; as a result, subjects who ate them consumed less energy than those who ate muffins without added pomace [39]. A study of chokeberry pomace as an addition to shortcrust pastry demonstrated a similar effect. The study aimed, among other things, to assess the inhibitory effects of shortcrust cookies containing various proportions of chokeberry pomace on the enzymes α-amylase, α-glucosidase, and lipase. The α-amylase inhibitory effect ranged from <0.50 mg/mL (30 and 50% addition of chokeberry pomace) to 221.76 mg/mL (control cookies). The inhibition ability of the shortcrust pastries without pomace was more than 400 times lower than that of the cakes with 50% pomace. Lipase inhibitory activity ranged from 9.12 mg/mL (50% addition of chokeberry pomace) to 16.76 mg/mL (control cookies). The study identified specific polyphenolic compounds, such as cyanidin-3-*O*-galactoside, cyanidin-3-*O*-glucoside, quercetin-3-*O*-galactoside, and others, that had a major impact on the ability to inhibit α-amylase and α-glucosidase. Moreover, it was shown that polyphenolic compounds in cookies with added pomace had a significant inhibitory effect on lipase activity [13].

The above research suggests that consuming products containing fruit pomace may help control postprandial glucose spikes not only in healthy people but also in people who are overweight and at risk of metabolic diseases. This may be a promising path toward the development of functional foods that increase the consumption of dietary fiber and a new strategy for the food industry to produce food with a reduced glycemic index.

### 3.3. Anti-Inflammatory Properties

Research results show that consuming fruit pomace or extracts obtained from them reduces inflammation markers and the effects of oxidative stress. A study by Fernandez-Fernandez et al. (2021) analyzed the bioactivity of antioxidants, anti-inflammatory compounds, and carboidase inhibitors from raw citrus pomace and their bioactive fractions. Research shows that orange pomace contains anti-inflammatory compounds, such as nobiletin, hesperidin, and tangeretin, which can inhibit the production of nitric oxide (NO). These compounds may be responsible for the ability of citrus pomace to exhibit anti-inflammatory effects [40]. In other studies, nobiletin, which is the main flavonoid present in tangerines, was reported to be able to regulate the expression of pro-inflammatory cytokines such as TNF-α, IL-1β, and IL-6 [41].

Mohammadi et al. (2024) investigated the antioxidant and anti-inflammatory properties of apple phenolic compounds. The purification process itself increased the content of total phenolic compounds, flavonoids, and tannins by 3.35-, 40.31-, and 8.87-fold, respectively. The main phenolic compounds identified in the purified extract were flavonoids, chlorogenic acid, and hyperoside. The effect of the raw and purified extract on the oxidation of body fluids, erythrocytes, and the expression of cytokines in macrophages was examined. The results indicated that the purified extract had a stronger anti-inflammatory effect than the crude extract, acting mainly by stimulating HO-1 gene expression and inhibiting the production of inflammatory cytokines. The study highlights the potential to purify raw apple waste extract to increase the content of valuable antioxidants and anti-inflammatory compounds. The results showed that the purified extract significantly inhibited the LPS-induced NO release in RAW264.7 macrophage cells compared to the crude extract in a dose-dependent manner. By contrast, the crude extract did not suppress NO production at the tested concentrations. The effect of the extracts on the mRNA expression of pro-inflammatory cytokines in LPS-stimulated macrophages was also assessed, and it was shown that the culture with the purified extract had a more significant inhibitory effect than the use of raw extracts on the mRNA expression level of IL-1β, IL-6, and IL-10, in a dose-dependent manner [42].

A 3-month intervention experiment examined the effect of consuming beef burgers enriched with grape pomace flour on blood biochemical parameters and oxidative stress biomarkers. The intervention involved 27 men diagnosed with metabolic syndrome who ate a burger with pomace flour (7% containing 3.5% of fiber and 1.2 mg gallic equivalents (GE)/g of polyphenols) every day for one month, followed by a control burger for another month. The study showed that the consumption of the enriched burger with toppings led to a significant reduction in fasting glucose levels (from 89.3 to 87.3 mg/dL; *p* = 0.05), increased insulin sensitivity (from 3.2 to 2.7; *p* = 0.013), and increased plasma vitamin C levels (from 41.4 to 45.7 µmol/L; *p* = 0.010), and reduced levels of advanced protein oxidation products and the production of oxidized LDL lipoprotein (oxLDL) (from 234.5 to 196.0 ng/mL; *p* = 0.005). The results suggest that grape-pomace-based functional foods have the potential to control the risk of chronic diseases in humans [43].

Han et al. (2016) assessed the effect of the consumption of the combined ethanol extract from grape seeds and schisandra fruit on the improvement of biochemical parameters in patients with metabolic disorders and in overweight or obese people. The study involved 76 participants who were divided into a control group and groups with a low (grape pomace extract: 342.5 mg/day + omija fruit extract: 57.5 mg/day) and high dose of the extract (grape pomace extract: 685 mg/day] + omija fruit extract: 115 mg/day). After 10 weeks of supplementation, the group supplemented with high concentrations of the extract showed a significant improvement in the plasma lipid profile, including a reduction in total cholesterol (*p* = 0.04), LDL-cholesterol (*p* = 0.042), and non-HDL cholesterol (*p* = 0.026), and an increase in the concentration of apolipoprotein A-1 (from 2.82 to 2.98 ng/mL), a key component of HDL lipoprotein. Additionally, high-concentration supplementation reduced the atherogenicity index, interleukin-1b, and TNF-α and increased the antioxidant capacity of erythrocytes. The low concentration in the intervention also showed some positive effects, but the changes were not as significant as in the case of supplementation with high concentrations of the tested compounds. No significant changes were observed in body composition, nutrient intake, or hepatotoxicity indices [44].

The above research results indicate that fruit pomace of various origins can be a natural source of anti-inflammatory and anti-atherosclerotic compounds and create new possibilities in the treatment of obesity and its complications.

### 3.4. Antibacterial Properties

Fruit pomace is increasingly considered as a raw material for antibacterial use. This is due, among other things, to the high content of polyphenolic compounds such as anthocyanins, which are known for their anti-inflammatory, immunomodulatory, and antibacterial properties. Studies conducted on blackcurrant extract proved its promising antibacterial properties. The extract showed effective antibacterial activity against *Porphyromonas gingivalis*, *Aggregatibacter Actinomycetemcomitans*, *Actinomyces naeslundii*, and *Fusobacterium nucleatum* comparable to the 0.2% chlorhexidine solution used in standard oral hygiene fluids. This allows us to consider blackcurrant extracts as a means against periodontal disease associated with bacterial infections [45]. Polysaccharides from currant seeds (0.01 to 0.1% solutions of the isolated raw polysaccharide) showed anti-adhesive activity against *Helicobacter pylori* in the gastric mucosa. This is due to the high content of high-molecular-weight acidic galactans, which bind to receptors on the surface of bacteria [46]. Additionally, studies on rats have shown that anthocyanins from blackcurrant (the total anthocyanin content in blackcurrant extract was 32% (*w*/*w*), consisting of delphinidin-3-glucoside, delphinidin-3-rutinoside, cyanidin-3-glucoside, and cyanidin-3-rutinoside) can modify the intestinal bacterial microbiota, increasing the growth of beneficial intestinal bacteria from the group of *Bacteroides*, *Prevotella*, *Porphyromonas*, and *Lactobacillus* spp. and reducing the number of harmful bacteria, such as *Bifidobacterium* spp. and *Clostridium perfringens* [47].

Bobinaitė et al. (2020) assessed the chemical composition and biological activities of extracts obtained from rowan pomace, and focused on acetone, ethanol, and water pomace extracts. The extracts were shown to effectively inhibit the growth of Gram-positive bacteria, with the acetone extract showing the highest antibacterial activity. In addition, the water extract showed the highest antibacterial activity against specific bacterial strains. The most sensitive among Gram-positive bacteria to the extract were *B. cereus*, *B. subtilis*, and *E. faecalis*, while *P. aeruginosa* and *C. freundii* showed the highest sensitivity to the extracts among Gram-negative bacteria. The results suggest that rowan pomace extracts may be beneficial as sources of natural compounds with antibacterial properties [48].

Grape pomace extract obtained by enzymatic extraction showed strong antioxidant and antibacterial activity. Even at a concentration of 2%, the extract showed antibacterial activity against Gram-negative bacteria (*E. coli* and *P. aeruginosa*) and Gram-positive bacteria (*Staphylococcus aureus*—MSSA and MRSA). The antibacterial activity of grape extract is attributed to the synergistic effect of components such as minerals (sodium and potassium), anthocyanins, phenolic acids, and organic acids (acetic, citric, and tartaric), and xylooligosaccharides, known for their antibacterial activity. The digestion process affects the antibacterial potential. The digested extract lost activity against Gram-negative bacteria and showed a reduced effectiveness against Gram-positive strains compared to undigested grape extract. The results indicate the need to consider the method of extract administration, allowing the full antibacterial activity in the gastrointestinal tract to be maintained. The grape pomace extract also showed prebiotic potential, stimulating the growth of probiotic strains. The presence of glucose and xylooligosaccharides in the extract contributes to faster bacterial growth. The results confirm that the extract can work as a source of fermentable carbohydrates, promoting the growth of *Bifidobacterium* and *Lactobacillus* and the production of organic acids [49].

The effect of cranberry pomace extract on the growth and gene expression of *Salmonella enterica* serovar Typhimurium, Enteritidis, and Heidelberg strains was demonstrated. The results indicated that cranberry extracts, in particular, subfractions CRFa20 (anthocyanins) and CRFp85 (flavonols), showed antibacterial activity against the tested *Salmonella* isolates. The minimum inhibitory concentrations (MICs) and minimum bactericidal concentrations (MBCs) of these fractions were determined. The MIC and MBC values were 8 and 16 mg/mL, respectively, against all tested *Salmonella enterica* isolates. The MIC value was 4 mg/mL for both CRFa20 and CRFp85 subfractions, and a reduced MBC value was obtained for CRFp85 (4 mg/mL). The study also showed that cranberry extracts modulated the transcriptomic profile of *Salmonella* Enteritidis, causing changes in the expression of genes related to virulence, motility, and iron transport. Cranberry pomace extracts may be used to reduce the spread of *Salmonella* strains through food [50].

There is a growing demand for natural food preservatives, resulting from consumer preferences for minimally processed products with natural additives and changes towards innovative biopreservation concepts using natural antimicrobial substances [51]. The above research results allow the consideration of polyphenol-rich fruit pomace extracts as natural food preservatives with antibacterial activity.

## 4. The Use of Fruit Pomace in the Food Industry

Fruit pomace is a by-product of fruit processing. It includes peels, seeds, and pulp obtained after juice extraction. The large mass of pomace poses an economic and microbiological challenge. The commercial use of these types of by-products can provide both financial and health benefits. There are research results indicating successes in the management of waste from the agri-food industry, including in the production of health-promoting food products [14,25,52,53,54]. It should be emphasized that the type of pomace significantly influences its properties, including its nutritional value, antioxidant activity, and potential applications. For example, Krajewska et al. (2024) emphasize that different types of pomace exhibit different levels of phenolic compounds, which are crucial for their antioxidant properties [55]. Meena et al. (2022) also demonstrated that the fiber content and functional properties of pomace differ depending on the source, which affects its suitability for potential use in the food industry [56]. Gumul et al. (2023) also emphasize that the moisture content and oil absorption capacity vary depending on the type of pomace, which may affect their use in food products [57]. The type of pomace thus has a key role in determining its functional properties and potential applications, underscoring the need to tailor approaches to the use of pomace.

In their research, the authors use both fruit pomace and its extracts. Fruit pomace is most often a by-product of juice extraction and consists of the cell walls, seeds, and skin of the fruit [58]. Depending on the type of fruit, the composition, nutritional value, and properties of pomace vary. Most of them are characterized by a high content of polyphenolic compounds, high antioxidant activity, and high content of dietary fiber, but the shares of soluble and insoluble fractions are varied [59,60,61].

Researchers are designing functional food recipes with various uses of pomace as an additive. Most often, pomace is dried, crushed, and added to food products, for example, as a partial substitute for wheat flour. However, the form of preparation of pomace for the further processing of pomace may vary depending on the type of pomace and its purpose. For bread production, pomace preparation included: black chokeberry pomace—freezing and freeze-drying [62]; apple pomace—freezing (−25 °C), defrosting, washing in water (1:1 proportion *w*:*v*), drying (at 60 °C for 72 h) [63], or drying in a cabinet dryer at 58 °C [64]; grape pomace—freeze-drying to a moisture content of about 2–4% [65] or drying in a convection oven for 3 h at 80 °C or freezing at −24 °C, thawing, and drying in a dehydrator at 40 °C until the mass stabilizes [66]; lemon pomace: washing, removing seeds, and air-drying (at 65 °C for 24 h) [67]; pomegranate pomace—soaking at 75 °C for 10 min, squeezing out water, and drying with hot air at 75 °C for 8 h [68]; banana pomace—separation of the skins, immersion in 0.5% citric acid to prevent enzymatic blackening, and drying on trays at 35 °C [69]; mango pomace—blanching the cleaned skins and spreading them in thin layers on trays, then drying them at 60 ± 2 °C using a tray dryer for 18 h until a constant weight is obtained [70]; bignay berries pomace—freezing at −20 °C and grinding, drying in a convection oven (at 45 °C for 48 h), or freeze-drying was carried out at a heater temperature of 25–30 °C, chilling temperature of −30 °C, and operating vacuum pressure of 100–300 Pa for 30 h [71]; orange pomace—freeze-drying, grinding, and freezing at −20 °C [72].

In the case of the production of sweet snack products, the preparation of pomace also depended on its type: apple pomace—drying in a drying cabinet at 50 ± 2 °C for 24 h [9] or drying in an oven at 55 ± 2 °C for 8 h [73] or drying for 3 h in the oven (60 °C) [74] or drying at 60 °C to optimum moisture content (10%) [75] or blanching with boiling drinking water for 30 s, and drying on trays at 60 ± 2 °C for 48 h using a convection oven with air circulation [76]; cherry—freezing at −18 °C and then freeze-drying using the FreeZone freeze-drying system (at −51 ± 1 °C in a vacuum of 0.055–0.065 mbar for 48 h) [39]; grape—drying at 120 °C for 60 min in a ventilated oven [77] or storage in a refrigerator at 4 ± 1 °C until further drying for 2 months, then drying at 55 and 75 °C [78] or drying at 60 °C for 12 h [79]; pomegranate—drying in a drying cabinet at 50 °C, crushing, and storing at −20 °C until use [80]; watermelon and melon—drying at 50 °C for 24 h in an air oven [81]; pineapple, apple, and melon—storage at 18 °C for a maximum of 30 days, and freeze-drying (40 °C, 0.998 mbar, 96 h) [82].

In turn, the preparation of pomace for the production of extruded products included: apple pomace—drying at 50 °C for 12 h in an oven with air circulation [83]; pineapple—freeze-drying for 72 h under vacuum conditions, grinding, and storing at −20 °C for further analysis [84]; mango and papaya—freezing (−30 °C for 24 h), then freeze-drying at −50 °C for 72 h, grinding, and storing at −30 °C until further use [85].

In addition to the direct use of fruit pomace, extracts are also used. Considering the occurrence of oxidation reactions of polyphenolic compounds, polyphenols can also occur as components attached to cell wall polysaccharides [86], which may influence the antioxidant and antiviral properties attributed to fruit pomace extracts [87]. The functional ingredients most commonly extracted from fruit pomace are: pectin, phenol, and dietary fiber. Due to their high nutrient concentration, these extracts may play a more obvious role in food products such as cookies [88], ciders [89], and meat products [90]. Currently, the most commonly used pectin extraction methods include enzymatic [91], mechanical [92], and chemical extraction [93]. New methods are constantly being proposed. For example, Wang et al. (2014) extracted pectin from apple pomace by adding subcritical water 30 times. The mixture was heated at 150 °C for 5 min, the supernatant liquid was filtered, and the pectin product was collected by alcohol precipitation, washing, and drying. The pectin recovery rate was 16.68% [94]. Current phenol extraction methods include maceration and Soxhlet extraction [95], the enzymatic method [96], ultrasonic extraction [97], and microwave extraction [98]. In turn, dietary fiber extract is obtained by acid–base digestion [99].

Additionally, when assessing the possibility of using fruit pomace in the food industry, the influence of temperature on the content of polyphenolic compounds should be taken into account, especially in the case of baked products. In the study by Górnaś et al. (2016), the stability of polyphenolic compounds in muffins enriched with strawberry, blackcurrant, raspberry, and cherry pomace (50 g/kg) was assessed, baked at three different temperatures (140, 180, and 220 °C) in a conventional and halogen oven. The most unstable compounds were anthocyanins (36–97% reduction). The concentration of individual anthocyanins decreased with increasing baking time, except for muffins prepared at 140 °C (in a traditional oven, for 35 min). This suggests that not only the baking time and temperature but also the heating source have a significant influence on the stability of anthocyanins during baking [100]. Anthocyanins are sensitive to UV radiation [101]. The most stable were flavonol glycosides (reduction by 0–21%). A more than 100% recovery was observed for neochlorogenic acid (95–120%) and ellagic acid (341–823%). The increase in free ellagic acid content in muffins enriched with strawberries or raspberries was positively correlated with baking time (r = 0.879 and r = 0.944, respectively) and was the result of the thermal hydrolysis of ellagitannins and ellagic acid glycosides. Due to the diverse composition of polyphenols in different fruits and the possibility of the hydrolysis of large molecules into monomolecules (for example, ellagitannins into ellagic acid) during the baking process, each case should be considered individually to obtain the optimal nutritional effect of baked goods enriched with fruit pomace [100,102].

It should be noted that the use of fruit pomace is the subject of research by many scientists. However, there are few products available on the market with the addition of these functional ingredients. These include, among others: Amazon Brand—Solimo Pomace Olive Oil, Indus Pomace Blend Olive Oil, Casa Emma Grape Pomace Flour, Cavalier Equestrian INC Apple Snacks, and Paté de Ciervo Venison paté in Potes pomace. The potential use of fruit pomace and its extracts is in the production of bread, sweet snack products, and extruded snacks.

### 4.1. Bread

Recipe ingredients for bread should be composed in such a way as to obtain a product with the appropriate nutritional value, technological parameters, and sensory values. There are research results available in the literature determining the impact of adding fruit pomace to bread. In all cases, their positive impact on health-promoting properties, especially on the increase in dietary fiber content, was demonstrated [62,63,65,67,68,69,70,103,104]. Dietary fiber has many important functions, including a beneficial effect on the prevention and treatment of chronic diseases, including diabetes, neurodegenerative diseases, cardiovascular diseases, and cancer. A rich supply of dietary fiber increases the feeling of satiety and promotes weight loss. Additionally, it modulates the intestinal microbiome, increases intestinal immunity and integrity, and promotes the adhesion of probiotic bacterial strains [105,106,107,108]. Enriching the bread recipe with fruit pomace was also associated with an increase in the content of minerals. The addition of apple pomace (0, 5, 6, and 8%) to gluten-free bread recipes increased the content of minerals, especially Cu (from 0.00–0% to 0.05 mg/100 g—8% pomace), Mg (from 0.00–0% to 3.78 mg/100 g—8% pomace), Mn (from 0.00–0% to 0.03 mg/100 g—8% pomace), and Fe (from 0.00–0% to 0.11 mg/100 g—8% pomace) [63]. Furthermore, bread enriched with chokeberry pomace (0, 1, 2, 3, 4, 5, and 6%) was characterized by a higher content of minerals, expressed as a higher ash content (from 0.928 ± 0.003–0% pomace to 0.974 ± 0.003%–6% pomace) [62]. Another important ingredient in bread enriched with residues of the fruit industry are polyphenolic compounds, which, despite thermal treatment, are still present in the finished products [62,63]. It has been shown that the effect of baking on the content of polyphenol compounds depends on factors such as temperature, time, and type of food product. The addition of passionfruit peel powder (from 5 to 20%) to bread resulted in an increase in the content of polyphenolic compounds, with the highest level shown at a substitution rate of 20%. The polyphenol contents in bread samples were 1355 mg/kg, 1637 mg/kg, 1773 mg/kg, and 1838 mg/kg for the substitution rate of 5%, 10%, 15%, and 20%, respectively [109]. Similarly, the use of grape pomace powder in bread resulted in an increase in the content of phenolic compounds, especially anthocyanins, the highest content of which was recorded at a substitution coefficient of 10 g/100 g [110]. Enriching wheat bread with grape pomace powder (0, 5, and 10%) also resulted in a higher content of phenolic compounds, especially anthocyanins, as well as increased antioxidant activity. Regarding the fortified bread, the greatest (*p* < 0.05) content in phenolic compounds was recorded for the 10% addition of pomace (considering both bound and free fractions), at 127.76 mg/100 g dry matter, followed for the 5% addition (106.96 mg/100 g dry matter), and control sample (63.76 mg/100 g dry matter). The use of grape pomace determined an increase in anthocyanins, recording 20.98 mg/100 g dry matter for the 5% addition of pomace and 35.82 mg/100 g dry matter for the 10% addition of pomace. The bioaccessibility of anthocyanins increased in both the 5% and 10% breads when moving from the gastric to the small intestine in vitro digestion phase with an average value of 24% [62,111]. Gluten-free bread enriched with apple pomace (0, 5, 10, and 15%) was also characterized by a higher content of phenolic compounds (from 1.02 ± 0.00 mg gallic acid/100 g dry matter—control sample to 21.96 ± 2.00 mg gallic acid/100 g dry matter—15% addition of pomace), including total phenols, flavonoids, phenolic acids, and phloridzin [63,112].

In addition to its nutritional value, the addition of fruit pomace to bread dough affects its rheological properties. Most of the studies focused on the impact of adding pomace to bread without the prior optimization of its properties. Some authors focused on the prior preparation of pomace, including by the appropriate time and type of drying or grinding of these waste products. Various drying techniques, such as microwave drying, oven drying, freeze drying, and air drying, were investigated to determine their effects on the physicochemical, functional, and bioactive properties of fruit pomace. Microwave drying was found to improve the quality characteristics of apple pomace powder, including the total phenolic content and antioxidant activity [113]. Additionally, freeze-drying has been shown to result in a higher phenolic and anthocyanin content and greater antioxidant properties compared to convection drying for bignay pomace (*Antidesma bunius*) [71]. In turn, drying red grape pomace at high temperatures reduces the content of polyphenols and anthocyanins, as well as the antioxidant activity of extracts from these pomaces [114]. Drying grape pomace preserves valuable and biologically active compounds while reducing microbial contamination [115]. Additionally, the enzymatic treatment of fruit pomace before drying improves the functional properties of the final powders in terms of dietary fiber composition and antioxidant release [116]. It has been shown that thermal and mechanical stresses occurring during the extrusion of apple pomace affect the composition and structure of dietary fiber, as well as the functional properties of the pomace [117]. The rheological properties of the dough resulting from the addition of fruit pomace are related to the interactions between the pomace ingredients. Additionally, pomace particles can interact with the structure of the gluten mesh. One of the studies focused on the addition of freeze-dried and powdered chokeberry pomace (0, 1, 2, 3, 4, 5, and 6%) to wheat bread. It was shown that the addition of these waste products increased the water absorption of flour but resulted in the reduced stability and weakening of the dough. It was observed that, after baking, the volume of the bread decreased (from 349.0 ± 4.2—control sample to 237.9 ± 4.7 cm^3^/100 g for 6% of pomace), while the hardness of the crumb increased (from 7.77 ± 0.30—control sample to 21.36 ± 0.65 N for 6% of pomace) [62]. Šporin et al. (2017) also documented the adverse effect of by-products on the volume of bread. The authors observed a negative impact of grape pomace powder on this parameter, which decreased significantly with the increase in their share (0, 6, 10, and 15%) [118]. Similarly, Torbica et al. (2019) used 10% apple pomace powder in a whole-grain bread recipe and showed a significantly lower volume (1.864 ± 0.008 mL/g) of enriched bread samples compared to the control product (1.994 ± 0.008 mL/g) [119]. Breads enriched with pomace also have more favorable physicochemical properties, such as increased water absorption and reduced crumb hardness. Moreover, it was found that the pre-hydration of pomace in hot water (for 30 min) improved the functional properties of the dough and the quality parameters of the resulting bread [120]. Excessive amounts of dietary fiber from fruit pomace may damage the aerated structure of bread. This is caused by the dilution of gluten, interaction of gluten with the fibrous material of pomace, disruption of the gluten structure, and competition for water between fiber and gluten protein, which leaves insufficient water to form the gluten mesh [66,72,121,122,123,124]. The reduction in volume and increase in firmness of bread loaves resulting from the addition of fruit ingredients may also be caused by the reduction in the amount of gluten in the dough as a result of the partial replacement of wheat flour with these products. Since one of the main functions of the gluten network is to retain gases during fermentation, a lower gluten content can lead to a smaller bread volume. The addition of dried and crushed apple peels, a by-product resulting from the production of juices, has a significant impact on the stability and extensibility of the dough, reducing the hardness of the finished product. The results of the image analysis also showed that the color of the crust of bread with the addition of pomace was darker compared to that of the control product [125,126].

In addition to the technological and qualitative features of bread, an important distinguishing feature when designing bread recipes is their sensory properties. Based on the opinions of potential consumers, it is possible to determine the maximum doses of health-promoting additives that they are willing to accept. Phytochemicals contained in fruits can significantly affect the color, aroma, and taste of food products to which they are added [127]. Studies show that too large an addition of by-products from the fruit industry negatively affects the sensory qualities of cereal products, especially those of bread [128]. In turn, other studies emphasized that the addition of pomace at a maximum of 3% is completely acceptable to consumers compared to the control sample. This addition also had a positive effect on the aroma and consistency of the bread [64]. The acceptance of a higher share of apple pomace (at the level of 10%) in wheat bread was confirmed by the research of Valková et al. (2022) [129]. The use of fruit pomace in bread production is presented in Table 1.

### 4.2. Sweet Snack Products

Sweet snack products are among the widely known and consumed products around the world. In the available literature, there are reports demonstrating the possibility of using fruit pomace to make cakes. The maximum addition of fruit pomace to sweet products is higher than in the case of bread dough and is above 30%. The possibility of a higher share of waste products in this type of products results, among other things, from the fact that other ingredients are also added to confectionery products (mainly sucrose or other sweeteners and fat), which mask the bitter taste of pomace. Additionally, in these doughs, the gluten mesh does not play such an important role as in the case of bread dough, so a higher share of gluten-free additives can be used. In the literature, the confectionery products whose recipes most often involve the partial substitution of flour with pomace are muffins. These products are the least sensitive to the addition of waste products and to the particle size of various ingredients. The most frequently used waste from the fruit and vegetable industry is apple pomace [73,74], but other types of fruit pomace are also used, such as mango [131,132], grape [127], cherry [39], and berry [133].

Similarly to the case of bread dough, enriching sweet snack recipes with fruit pomace resulted in an increase in the content of dietary fiber (especially the insoluble fraction), polyphenolic compounds, and minerals. The influence of the addition of pomace from various fruits, such as grapes, apples, and pomegranates, among others, was tested [9,74,77,78,80,134,135]. Specifically, the addition of chokeberry pomace at 50% (by weight of wheat flour) to a shortbread cookies recipe was shown to increase the dietary fiber content by 10 times (from 2.79 ± 0.14—control sample to 29.67 ± 0.03 g/100 g—cookies with 50% pomace), as well as the content of potassium (from 68.13 ± 1.63—control sample to 98.78 ± 3.26 mg/100 g—cookies with 50% pomace), calcium (from 40.66 ± 0.89—control sample to 110.58 ± 1.27 mg/100 g—cookies with 50% pomace), magnesium (from 19.81 ± 0.28—control sample to 25.77 ± 0.62 mg/100 g—cookies with 50% pomace), iron (from 1.28 ± 0.16—control sample to 9.53 ± 0.13mg/100 g—cookies with 50% pomace), anthocyanins, flavonols, phenolic acids, and flavan-3-ols (total polyphenol content ranged from 0.00—control sample to 807.6 ± 149.6 mg/kg—cookies with 50% pomace) [13]. Usman et al. (2020) found a reduced protein content (5.34 ± 0.70%) and an increased ash content (0.62 ± 0.02%) in cookies with increasing levels (0%, 5%, 10%, 15%, and 20%) of pomace-supplemented apple compared to the control sample (6.59 ± 0.47% and 0.45 ± 0.03%, respectively) [136].

The type of cakes in which the use of fruit pomace is limited are sponge cakes and sponge-fat cakes. The basic ingredients in these cakes are flour, sugar, eggs, fat, and leavening agents. Each of these products has a specific function. This should be considered when attempting to replace them with fruit pomace. One of the limitations resulting from the introduction of fruit pomace to the dough recipe is the negative impact of fiber on the formation of an aerated structure. Dietary fiber from fruit pomace has a high affinity for water, so its interaction with water is one of the main problems. The partial replacement of wheat flour with fruit pomace reduced the increase in dough volume, and the final products became more rubbery and less consistent [66,81,127,132,137,138,139]. The study by Goranova and Manev (2022) led to different results, which showed that the addition of apple pomace in a specific concentration has a significant impact on the texture as well as the moisture and fiber content of sponge cakes. Following the addition of 15% and 25% apple pomace, the textural properties (breaking force, breaking energy, hardness, gumminess, and compressive strength) decreased, and the strain at break, adhesion, and viscosity increased compared to the control sample. The share of 50% apple pomace in the recipe resulted in a significant increase in all textural properties, except for deformations caused by cracking [75]. Similarly, Sudha et al. (2007) observed a gradual reduction in cake volume when enriched with apple pomace at the levels of 10%, 20%, and 30%. Researchers observed a 27.06% reduction in the volume of the dough enriched with 30% apple pomace compared to the control product [140]. Hosseini and Pazhouhandeh (2023) showed that, as the addition of apple pomace (10, 20, and 30%) to cookies increased, the hardness (from 1.61 ± 0.06—control cakes to 6.24 ± 0.13 N—cakes with 30% pomace), gumminess (from 1.29 ± 0.05—control cakes to 4.48 ± 0.16 N—cakes with 30% pomace), and chewiness (from 1.31 ± 0.11—control cakes to 4.44 ± 0.10 N.mm—cakes with 30% pomace) of the products increased [74].

In the recipe for confectionery products, texture is one of the sensory characteristics that is most influenced by the content of dietary fiber. A meta-analysis of sensory studies showed that the fiber enrichment of baked cereal products reduces the acceptability of their texture, but the overall acceptability is high [141]. It was also shown that the addition of apple pomace at a maximum level of 10% to the cookie recipe showed no negative impact on the sensory characteristics [74]. In this regard, Alongi et al. (2019) also did not find significant changes in the sensory properties of cookies in which wheat flour was replaced by 8% to 14% with the addition of apple pomace powder [52]. Based on sensory and compositional attributes, Usman et al. (2020) also found that cookies with a good quality and better organoleptic properties could be prepared using 10% apple pomace [136]. Unfavorable changes in the sensory properties of cereal products were observed when a larger amount (15%) of apple pomace powder was used. This concerned every distinguishing feature of the sensory evaluation [82]. The use of fruit pomace in the production of sweet snack products is presented in Table 2.

### 4.3. Extruded Snacks

Extruded snacks are popular food products produced in a short time by extrusion at high temperature. The basis of this type of product is usually corn, but research results show that it is possible to use other additives to improve the nutritional value and organoleptic properties. The additives used to partially replace starch are, for example, fruit pomace [145,146,147,148]. The high content of dietary fiber in fruit pomace affects the following properties of the final products: the water solubility index, water absorption index, texture, crunchiness, expansion, starch digestibility, and sensory properties. During processing, raw materials are exposed to thermomechanical stresses that can cause physical and chemical changes in dietary fiber, especially the transition from insoluble to soluble high-molecular-weight dietary fiber. In turn, the content of polyphenolic compounds is reduced during the extrusion process [149,150,151,152]. Research has shown that the use of cherry, blackcurrant, and chokeberry pomace (5, 10, and 20%) as an addition to the recipe of extruded corn snacks resulted in an increase in the content of bioactive compounds (total phenolic ranged from 1.19 ± 0.37—control sample to 13.44 ± 0.43 mg catechin/g dry matter—extrudates with 20% chokeberry pomace and 4.97 ± 0.02 mg catechin/g dry matter—extrudates with 20% cherry pomace and 5.93 ± 0.03 mg catechin/g dry matter—extrudates with 20% blackcurrant pomace) and dietary fiber (from 0.95 g/100 g dry matter—control sample to 10.79 g/100 g dry matter—extrudates with 20% chokeberry pomace and 8.12 g/100 g dry matter—extrudates with 20% cherry pomace and 10.29 g/100 g dry matter—extrudates with 20% blackcurrant pomace) [57]. Despite the fact that extrusion can reduce the content of polyphenolic compounds by up to 46% (due to the partial depolymerization, decarboxylation, and polymerization of phenolic compounds and tannins) [153,154], it was shown that the addition of chokeberry pomace at the level of 20% significantly increased the content of total phenolic compounds, phenolic acids (from 0.255 ± 0.000—control sample to 0.590 ± 0.032 mg ferulic acid/g dry mater—extrudates with 20% chokeberry pomace), flavonoids (from 0.266 ± 0.021—control sample to 1.75 ± 0.057 mg rutin/g dry matter—extrudates with 20% chokeberry pomace), flavonols (from 0.200 ± 0.000—control sample to 0.389 ± 0.022 mg quercetin/g dry matter—extrudates with 20% chokeberry pomace), and anthocyanins (from 0.068 ± 0.011—control sample to 0.761 ± 0.034 mg cyanidin-3-glucoside/g dry matter—extrudates with 20% chokeberry pomace) in the snacks [57]. In turn, Schmid et al. (2021) showed that the extrusion treatment of chokeberry pomace resulted in a 75% reduction in the total anthocyanin content. The addition of starch during the extrusion process reduces the extrusion temperature by approximately 10 °C, but, despite this, the total anthocyanin content in the mixture of starch and pomace did not differ significantly from the anthocyanin content in extruded chokeberry pomace [151]. The degradation of anthocyanins to a similar extent has also been reported by other authors in relation to the extrusion processing of products derived from blueberry, cranberry, and raspberry [145,155,156]. When apple pomace was used, the content of protein, ash, fiber, calcium, potassium, and polyphenolic compounds in the enriched snacks increased, and this increase is proportional to the percentage of pomace (10 and 20%) [83].

Knowledge of the stability of dietary fiber and polyphenolic compounds is one of the factors for the proper enrichment of products extruded with fruit pomace. It is also important to optimize processing conditions in order to obtain final products with specific technical, physical, and sensory characteristics. Dietary fiber has an adverse effect on specific features of extruded products: solubility in water, water absorption, cross-sectional and longitudinal expansion, hardness, and color [148,151,157]. The addition of chokeberry pomace at a level above 25% reduces the crispiness of extruded products. Additionally, an increase in water content to 23% resulted in the formation of smaller, spherical pore cells, which resulted in an increase in the hardness of the products. However, this effect decreased with the increasing share of pomace [158]. In the case of apple pomace, other authors also showed a reduction in pore size and an increase in the thickness of cell walls [146]. The hardness of extruded products decreases as the starch content increases. The addition of pomace inhibits expansion and the porous structure, thereby reducing the hardness (from 97 ± 32—control sample to 20 ± 8 Nm^−2^—extrudates with 50% pomace) of the final products [147,158]. Extrudates based on corn flour and apple pomace showed significantly higher crushing forces (with the addition of pomace from 17% to 28%, the thickness of the cell walls was reduced by 32–44% and the average diameter of the cell wall by 62%) [146]. Such dependencies were not demonstrated in the case of the addition of pineapple pomace [84]. In turn, Gumul et al. (2023) proved that the addition of chokeberry pomace at the level of 20% did not worsen the physical properties (water-binding capacity, hardness, and expansion coefficient) of gluten-free extruded snacks, which affects the quality of the obtained product. Therefore, such snacks could be recommended for commercial production to increase the availability of gluten-free products for people with celiac disease [57].

The level of pomace addition and the water content primarily affect the color of the extruded products: the higher the pomace content, the more intense the color [158,159]. The extrusion process itself changes the color, but the use of chokeberry pomace maintains an attractive purple color [158]. The use of fruit pomace is used as a partial substitute for flour in extruded products, leading to a lower starch content, which decreases water absorption, affecting starch gelatinization. Additionally, dietary fiber and starch compete for water, which affects the water solubility index [160]. The high affinity of dietary fiber for water results in no differences in the water solubility index in the case of products extruded from corn flour and products enriched with pineapple, orange, or grape pomace [84,161]. The use of fruit pomace in extruded products also contributes to an increase in the bulk density of finished products and a reduction in radial expansion (by up to 57%). This is due to a lower extensibility and gas-holding capacity [84,146,162,163]. The use of fruit pomace for the production of extruded products usually produces products with a sweet and fruity flavor [164,165]. It was shown that the most favorable sensory sensations were caused by the use of a 10% addition of defatted blackcurrant seeds, while a higher addition of 30% significantly reduced the overall consumer acceptability [166]. It has also been proven that the method of processing waste products from the fruit industry affects sensory properties. It was shown that extrudates with blackcurrant pomace from non-enzymatic press residues received higher ratings from sensory panel participants than extrudates with blackcurrant pomace, which were subjected to conventional enzymatic treatment before pressing [167]. The use of fruit pomace in the production of extruded snacks is presented in Table 3.

## 5. Conclusions

Fruit pomace is a valuable by-product due to the significant content of bioactive compounds and is still underutilized in the food industry, among others. Due to their large amount produced mainly after juice extraction, their improper and insufficient management poses a threat to the environment and even public health. Based on many studies, it has been confirmed that organic compounds (mainly from the polyphenol group) contained in fruit pomace can have a beneficial effect on the functioning of the body through their antioxidant, antidiabetic, anti-inflammatory, and antibacterial effects. In addition to the health-promoting properties of fruit pomace, this review discusses the possibilities of their use in the food industry (bread, sweet snack products, and extruded snacks). The research results indicate that the addition of fruit pomace to food products may be a valuable strategy for modulating not only the health-promoting properties but also the technological and sensory properties of the final products. However, the final effect depends on both the type of pomace and its share in the product recipe. It should be emphasized that there are few works in the available literature that provide a multi-faceted analysis of the possibility of using fruit pomace in specific food products, considering health-promoting properties and nutritional value, as well as technological and sensory features. Therefore, further comprehensive research is necessary to accelerate the efficient use of fruit industry waste products in the large-scale production of functional foods.

## Figures and Tables

**Table 1:** Use of fruit pomace in bread production.

Type of Pomace	Type of Food Product	Addition of Pomace [%]	Type of Research	Obtained Technological Effect	Obtained Health Effect	Sensory Evaluation	References
grape	breadstick	5 and 10	physicochemical, nutritional value, and sensory analysis	↑water absorption ↑tenacity ↓extensibility ↓swelling index ↓deformation energy ↓hardness ↓fracturability ↓volume	↑DF ↑TPC ↑AA (FRAP and ABTS)	↑odor ↑acidity ↑bitterness ↑astringency ↑hardness ↓regularity of alveolation ↓friability	[130]
grape	bread	4, 6, 8, and 10	nutritional value and sensory analysis	not tested	↑TPC ↑DF ↑AA(FRAP) ↓TC ↑HDLc ↓LDLc ↓glucose ↓leptin	↓taste ↓volume ↑hardness ↓typical aroma maximum addition—6%	[65]
grape	bread	2, 5, and 10	physicochemical and sensory analysis	↓specific volume ↑hardness	↑TPC ↑AA (DPPH)	maximum addition—5%	[104]
grape	wheat based bread	5 and 10	chemical analysis	not tested	↑TPC ↑anthocyanins ↑AA (FRAP and ABTS) ↓predicted GI	not tested	[111]
grape	wheat bread	6, 10, and 15	physicochemical and sensory analysis	↑firmness ↓volume	↑TPC ↑AA (DPPH and FRAP)	↓springiness ↓toughness ↓hardness ↑crumbliness ↑adhesivity ↑sand feeling	[118]
grape	bread	5, 10, and 15	physicochemical and sensory analysis	↓volume ↑chewiness ↑firmness	↑TPC ↑AA (DPPH) ↑DF	maximum addition—10%	[66]
grape	wheat bread	1, 2, 5, and 8	physicochemical and sensory analysis	↓volume ↑dough stability	↑TPC ↑minerals	the most favorable addition—1%	[125]
black chokeberry	wheat bread	1, 2, 3, 4, 5, and 6	physicochemical and sensory analysis	↑water absorption ↑crumb hardness ↓stability and weakening of the dough ↓volume	↑minerals ↑DF ↑TPC ↑AA	maximum addition—3%	[62]
apple	gluten-free bread	5, 6, and 8	physicochemical and nutritional value analysis	↓hardness ↓chewiness ↓cohesiveness ↓springiness ↓resilience	↑DF ↑minerals	not tested	[63]
apple	gluten-free bread	5, 10, and 15	chemical and sensory analysis	not tested	↑TPC ↑total flavonoids ↑AA (TEAC)	↓color ↓elasticity of the bread ↓crumb porosity ↑taste ↑smell maximum addition—5%	[112]
apple	wholegrain wheat bread	10 and 20	physicochemical and nutritional value analysis	↓volume	↓protein ↓fat	acceptable by consumers	[119]
apple	sangak bread	1, 3, 5, and 7	rheological tests and sensory analysis	↓cohesiveness	not tested	maximum addition—3%	[64]
apple	wheat bread	1, 2, 5, and 10	physicochemical, nutritional value, and sensory analysis	↓volume	↑ash ↓protein ↓fat ↑carbohydrates ↓energy value ↑TPC ↑AA	acceptable by consumers	[129]
banana	wheat bread	10	physicochemical, nutritional value, and sensory analysis	↓specific volume ↑density ↓loaf height	↑ash ↓protein ↓fat ↑crude fiber ↓carbohydrate ↑DF ↑TPC ↑AA (DPPH and FRAP)	↓color other distinctions acceptable	[103]
banana	chapatti (unleavened Indian flat bread)	5, 10, 15, and 20	physicochemicaland sensory analysis	↑subjective score in kneading and ↑rollability ↑dough stickiness ↑dough strength ↓tear force	↑TPC ↑flavonoid ↑AA (DPPH)	↓color ↓texture ↓taste ↓overall acceptability	[69]
lemon	steamed bread	3 and 6	physicochemical analysis	↑stiffer ↓extensible ↑hardness ↓cohesiveness ↓specific volume ↓elasticity	↑TPC ↑AA	not tested	[67]
pomegranate	bread	5 and 15	physicochemical and nutritional value analysis	not tested	↑DF ↓carbohydrate ↑minerals ↑TPC ↑AA (DPPH)	↓appearance ↓color ↓taste ↑flavor ↓mouth feel ↓overall acceptability	[68]
mango	whole wheat bread	0, 1, 3, and 5	physicochemicaland sensory analysis	↑viscoelastic property ↓loaf height ↓weight loss percentage ↓specific volume ↑bread density ↑crumb moisture ↑brownness index ↑hardness, ↑cohesiveness ↑springiness	↑TPC ↑AA (FRAP and DPPH)	↓porosity ↓traditional bread aroma ↑fruity aroma ↓fruity taste ↑after taste ↑crumb color ↑hardness ↑stickiness	[70]
passiflora edulis	bread	5, 10, 15, and 20	physicochemical, nutritional value, and sensory analysis	volume ↑specific gravity ↑cohesiveness ↓springiness ↑hardness	↑energy value ↑DF ↑TPC	↓overall acceptability maximum addition—10%	[109]
blackcurrant	wheat bread	10	not tested	↓volume ↑dough ↓resistance ↓extensibility ↓dough stickiness ↓dough pH ↓bread pH ↓bread volume ↑crumb moisture ↑crumb cell density ↑total cell area	not tested	not tested	[120]
orange	gluten-free bread	5.5	physical and sensory analysis	↑robustness ↓starch gelatinization	not tested	acceptable by consumers	[122]

Abbreviations: AA, antioxidant activity; ABTS, 2,2′-azino-bis-(3-ethylbenzothiazoline-6-sulfonic) acid; DF, dietary fiber; DPPH, 2,2-diphenyl-1-picrylhydrazyl; FRAP, ferric-reducing antioxidant power; GI, glycemic index; HDLc, high-density lipoprotein cholesterol; LDLc, low-density lipoprotein cholesterol; TC, total cholesterol; TEAC, trolox-equivalent antioxidant capacity; TPC, total phenolic compounds; ↑, increase/improvement; ↓, decrease/worsening.

**Table 2:** Use of fruit pomace in the production of sweet snack products.

Type of Pomace	Type of Food Product	Addition of Pomace [%]	Type of Research	Obtained Technological Effect	Obtained Health Effect	Sensory Evaluation	References
apple and orange	rice-flour-based gluten-free cake	0, 5, 10, and 15	physicochemical, nutritional value, and sensory analysis	↑viscosity ↑elastic ↑specific gravity ↑crumb hardness ↓specific volume of cakes	↑DF	highest overall acceptance at 5% additive (highest with orange pomace)	[142]
apple	short dough biscuit	10 and 20	nutritional value and sensory analysis	↓volume ↓hardness ↑browning of cakes	↑DF ↑TPC ↓GI	acceptable by consumers	[52]
apple	cookie	5, 10, and 15	physicochemical, nutritional value, and sensory analysis	↑water activity	↑DF	high consumer acceptance with the addition of 15%	[82]
						↓	
apple	bun	10, 15, and 20	physicochemicaland sensory analysis	↓volume ↓crust color ↓crumb color ↓grain ↑texture	↑free radical scavenging ↑cyto/DNA protective properties	maximum addition—15%	[73]
apple	muffin	10, 20, 30, and 40	physicochemicaland sensory analysis	↓volume ↓crust color ↓crumb color ↓grain ↓texture	↑free radical scavenging ↑cyto/DNA protective properties	maximum addition—30%	[73]
apple	cookie	10, 20, and 30	physicochemicaland sensory analysis	↓spread ↓surface cracking ↓crumb color ↓texture	↑free radical scavenging ↑cyto/DNA protective properties	maximum addition—20%	[73]
apple	cake	10, 20, and 30	physicochemical and sensory analysis	↑hardness ↓volume ↑density ↑gumminess ↑chewiness	↑DF	maximum addition—10%	[74]
apple	muffin	5, 10, 15, 20, 25, 30, 35, 40, 45, and 50	physicochemical, nutritional value, and sensory analysis	↑water holding capacity ↑fat absorption capacity ↑swelling power ↓foam capacity	↑ash ↑DF ↓crude protein ↓energy value	↓crust color ↓crumb color and softness ↓crumb structure ↑fruity flavor ↓texture maximum addition—33%	[9]
apple	cookie	5, 10, 15, 20, and 25	physicochemical, nutritional value, and sensory analysis	↓thickness ↑spread factor ↓color of cookies with color meter ↓falling number	↑ash ↑DF ↑TPC	↓taste ↓mouth-feel of cookies ↓overall acceptability maximum addition—10%	[136]
apple	muffin	4, 8, 16, 24, and 32	chemical and nutritional value analysis	not tested	↓crude protein ↑DF ↑TPC ↑AA (FRAP and ORAC)	not tested	[76]
apple	sponge cake	15, 25, and 50	physical and nutritional value analysis	↑all textural characteristics ↑moisture ↓stickiness	↑DF	not tested	[75]
apple	cake	5, 10, and 15	physical and nutritional value analysis	↑water absorption ↓dough stability ↑mixing tolerance index (weakening of the dough) ↑resistance to extension ↓in peak viscosity ↓volume	↑DF ↑TPC	not tested	[140]
grape	muffin	15 (with different particle size fractions)	physicochemical and sensory analysis	particle fragmentation had a negative effect on muffin hardness and lightness and pore homogeneity	↑ antioxidant compounds (regardless of particle size) ↑DF (regardless of particle size) ↑total anthocyanins ↑total phenol content ↑ AA (ABTS and DPPH, with a reduction in particle size)	↓acceptability as pomace fineness increases	[77]
grape	gluten-free muffin	15 and 25	physicochemical, nutritional value, and sensory analysis	↓muffin volume at a higher addition an increase in pomace content led to a change in texture, with a higher percentage resulting in a more granular texture	↑DF ↑protein	greater consumer acceptance with 15% addition	[78]
grape	cookie	2, 4, 6, and 8	physicochemical, nutritional value, and sensory analysis	↑browning of cakes	↑DF ↑protein ↑ash ↑anthocyanins ↑TPC ↑AA	maximum addition—6%	[79]
grape	muffin	10, 15, and 20	physicochemical and sensory analysis	↓volume ↓springiness ↑firmness	↑TPC ↑radical scavenging activity ↑DF	maximum addition—10%	[66]
grape	brownie	10, 15, 20, and 25	physicochemical and sensory analysis	↓volume ↑springiness	↑DF	maximum addition—15%	[66]
grape	vegan muffin	5 and 10	physicochemical, nutritional value, and sensory analysis	↓pH ↑spread ratio ↓volume	↑ash ↓total starch ↑DF ↑AA (FRAP and ABTS) ↑TPC	↓typical smell of even baked cake ↑wine and the fruity odor ↑taste acceptable by consumers	[134]
pomegranate	cookie	7.5	chemical analysis	not tested	↓ellagitannin bioavailability ↑bioavailability gallic acid, ellagic acid ↑TPC ↑AA ↑inhibitory activity of α-glucosidase, α-amylase and lipase	not tested	[143]
pomegranate	muffin	5, 10, and 15	physicochemical, nutritional value, and sensory analysis	↑apparent viscosity ↑hardness ↓springiness	↑DF ↑ total phenolics ↑Mg, Ca, and K ↑AA	↓crumb cell structure ↓crumb color ↓crust color ↓chewiness	[80]
pineapple	cookie	5, 10, and 15	physicochemical, nutritional value, and sensory analysis	↑water activity	↑DF	high consumer acceptance with an addition of 15%	[82]
melon	cookie	5, 10, and 15	physicochemical, nutritional value, and sensory analysis	↑water activity	↑ash ↑DF	high consumer acceptance with an addition of 10%	[82]
chokeberry	shortcrust pastry	10, 30, and 50	nutritional value and sensory analysis	not tested	↑DF ↓energy value ↑TPC ↑AA ↑inhibitory activity of α-glucosidase, α-amylase and lipase	↓acceptability with an increase in the percentage of pomace	[13]
blackcurrant	shortbread cookie	10, 30, and 50	nutritional value and sensory analysis	not tested	↑DF ↓energy value ↑TPC ↑AA ↑inhibitory activity of α-glucosidase, α-amylase and lipase	↓acceptability with an increase in the percentage of pomace	[14]
apple chokeberry blackcurrant	shortbread cookie	10, 30, and 50	nutritional value analysis	not tested	↑DF ↓GI	not tested	[54]
blackcurrant	gluten-free cookie	3.75	physicochemical, nutritional value, and sensory analysis	not tested	↑DF ↑TPC ↑AA	↓flavor	[144]
mango	muffin	50 and 75	physicochemical analysis	↑moisture ↓rate of starch hydrolysis	↑ash ↑DF ↓ total soluble carbohydrates ↓available starch ↑total soluble polyphenol ↑AA (DPPH and FRAP)	not tested	[131]
mango	muffin	25	physicochemicaland sensory analysis	↓volume ↑specific gravity ↓firmness	↑DF ↑ash ↑protein ↑lutein ↑β-carotene ↑TPC	↓shape and appearance ↓grain ↓texture ↓overall quality	[132]
cherry	muffin	10, 20, 30, and 40	chemical, nutritional value, and sensory analysis	not tested	↑ash ↑DF ↓available carbohydrates ↑AA (FCR and DPPH) ↑TPC ↓AUC for hunger ↑AUC for fullness ↑AUC for satisfaction ↓AUC for prospective full intake ↓IAUC for blood glucose response ↓energy intake at subsequent meal	↓color ↓appearance ↓texture ↓flavor ↓taste ↓overall acceptance	[39]
raspberry	muffin	10 and 20	physicochemicalanalysis	no significant impact	↑TPC	not tested	[133]
cranberry	muffin	10 and 20	physicochemicalanalysis	↑hardness ↑gumminess	↑TPC	not tested	[133]
watermelon	cake	2.5, 5, and 7.5 (as a replacement for wheat flour)	physicochemicaland sensory analysis	↓crumb—total color intensity	↑ash ↓protein ↑TPC	↓appearance ↓crust color ↓crumb color ↓crumb texture ↓taste ↓odor ↓overall acceptability maximum addition—5%	[81]
		5, 10, and 15 (as a replacement for fat)		↑weight ↓crumb—total color intensity	↑ash ↑TPC	maximum addition—10%	
melon	cake	2.5, 5, and 7.5 (as a replacement for wheat flour)	physicochemicaland sensory analysis	↓crumb—total color intensity	↑ash ↓protein ↑TPC	↓appearance ↓crust color ↓crumb color ↓crumb texture ↓taste ↓odor ↓overall acceptability maximum addition—5%	[81]
		5, 10, and 15 (as a replacement for fat)		↑weight ↓crumb—total color intensity	↑ash ↑TPC	maximum addition—10%	
orange	muffin	10 and 15	physicochemicaland sensory analysis	↓moisture	↑ash ↑DF ↑slowly digestible starch ↓resistant starch ↓GI	maximum addition—10%	[138]

Abbreviations: AA, antioxidant activity; ABTS, 2,2′-azino-bis-(3-ethylbenzothiazoline-6-sulfonic) acid; AUC, area under the curve; DF, dietary fiber; DPPH, 2,2-diphenyl-1-picrylhydrazyl; FCR, Folin–Ciocalteu reagent assay; FRAP, ferric-reducing antioxidant power; GI, glycemic index; IAUC, incremental area under the blood glucose response curve; ORAC, oxygen radical absorption capacity; TPC, total phenolic compounds; ↑, increase/improvement; ↓, decrease/worsening.

**Table 3:** Use of fruit pomace in the production of extruded snacks.

Type of Pomace	Type of Food Product	Addition of Pomace [%]	Type of Research	Obtained Technological Effect	Obtained Health Effect	Sensory Evaluation	References
mango	corn extrudate	15	chemical and sensory analysis	not tested	↑TPC ↑AA ↑bioavailability	↑color ↑flavor ↑texture ↑overall acceptability	[85]
papaya	corn extrudate	15	chemical and sensory analysis	not tested	↑carotenoid content ↑release of bioactive compounds and antioxidant capacity was highest during the intestinal stage	↑color	[85]
apple	expanded extrudate	17, 22, and 28	physical and nutritional value analysis	↓expansion ratio ↑specific length ↓average cell diameter ↑cumulative volume ↑average crushing force ↑crispness work ↑spatial frequency of ruptures	↑fat ↑DF	not tested	[146]
apple	snack	10 and 20	chemical, nutritional value, and sensory analysis	not tested	↑protein ↑ash ↑DF ↑Ca, K ↑TPC	↑overall acceptability consumer acceptance at 10 and 20%	[83]
apple	extruded apple pomace	100	physicochemical analysis	↑water solubility ↓oil holding capacity	↓total extractable polyphenols ↑flavanols ↑phenolic acids ↑dihydrochalcones ↑AA (ORAC)	not tested	[159]
apple	extruded product	17, 22, and 28	physical analysis	↓starch gelatinization ↓starch solubilization ↓expansion ↓starch digestibility ↑cell wall thickness/cell size ratio	not tested	not tested	[162]
apple	extruded snack	10, 15, and 20	chemical and sensory analysis	not tested	↑TPC ↑AA (ABTS)	↑consistency	[165]
cherry blackcurrant chokeberry	gluten-free snack	5, 10, and 20	physical and nutritional value analysis	↑density ↓water binding capacity	↑TPC ↑AA (ABTS) ↑DF ↑total sugar ↑phenolic acid ↑flavonoids ↑flavonols ↑anthocyanins	not tested	[57]
chokeberry	ready-to-eat texturized cereal	100	chemical and nutritional value analysis	not tested	↑DF ↑anthocyanin ↑phenolic acids ↑flavonols	↑color ↑visual impression ↑taste	[151]
chokeberry	ready-to-eat texturized cereal	25 and 50	physicochemical and nutritional value analysis	↑water solubility index ↓sectional expansion index ↓size of pore cells	↑DF ↓bioaccessible glucose ↑anthocyanins ↑phenolic acids ↑flavonols ↑TPC	not tested	[158]
cranberry	extruded product	30, 40, and 50	chemical analysis	not tested	↓anthocyanin ↑flavonols ↑AA (ORAC) ↑procyanidin monomers and dimers ↓procyanidin oligomers	not tested	[156]
cranberry blueberry grape apple	corn starch extrudate	5, 15, and 30	physicochemical analysis	↓expansion ratio	↑DF	not tested	[148]
pineapple	extruded product	10.5 and 21	physicochemical analysis	↓expansion ratio ↓luminosity ↑redness	↑DF	not tested	[84]
grape	extruded product	2, 6, 10, and 12.7	chemical analysis	not tested	↑AA (DPPH) ↑TPC ↑β-glucan	not tested	[160]
grape	extruded product	2, 6, 10, and 12.73	physical and sensory analysis	↓sectional expansion index ↑bulk density ↑peak force ↓crispiness ↓lightness	not tested	↓color ↑taste (sweetness) acceptable addition of 2 or 10%	[164]
rosehip	extruded snack	10, 15, and 20	chemical and sensory analysis	not tested	↑TPC ↑AA (ABTS)	↓shape and size ↓taste and smell ↑consistency	[165]
blackcurrant	extruded product	10, 30, and 50	physicochemical, nutritional value, and sensory analysis	↓jaggedness ↓breaking strength	↑protein ↑fat ↓carbohydrate ↑ash ↑DF ↑TPC ↑flavonoids ↑AA (TEAC)	maximum addition—10%	[166]
blackcurrant	extruded snack	30	physicochemical, nutritional value, and sensory analysis	↑expansion ↓hardness ↓density ↓redness ↓pH	↑fructose ↑glucose ↑fruit acids	↑texture ↑appearance ↑flavor	[167]

Abbreviations: AA, antioxidant activity; ABTS, 2,2′-azino-bis-(3-ethylbenzothiazoline-6-sulfonic) acid; DF, dietary fiber; DPPH, 2,2-diphenyl-1-picrylhydrazyl; ORAC, oxygen radical absorption capacity; TEAC, trolox-equivalent antioxidant capacity; TPC, total phenolic compounds; ↑, increase/improvement; ↓, decrease/worsening.
